# Single versus sequential culture medium: which is better at improving ongoing pregnancy rates? A systematic review and meta-analysis

**DOI:** 10.5935/1518-0557.20170045

**Published:** 2017

**Authors:** Felipe Dieamant, Claudia G. Petersen, Ana L. Mauri, Vanessa Comar, Marina Mattila, Laura D. Vagnini, Adriana Renzi, Bruna Petersen, Juliana Ricci, João Batista A. Oliveira, Ricardo L.R. Baruffi, Jose G. Franco Jr.

**Affiliations:** 1 Center for Human Reproduction Prof. Franco Jr, Ribeirão Preto, Brazil.; 2 Paulista Center for Diagnosis Research and Training, Ribeirão Preto, Brazil.

**Keywords:** meta-analysis, controlled clinical trials, randomized, embryo culture techniques, pregnancy

## Abstract

This study aimed to evaluate if single medium is better than sequential medium at improving ongoing pregnancy rates in patients undergoing assisted reproductive technology (ART) procedures. The data featured in this meta-analysis were extracted from four randomized controlled trials yielded from a systematic search carried out on electronic databases. The primary endpoint was ongoing pregnancy rate. Secondary endpoints included clinical pregnancy and miscarriage rates. The endpoints for ongoing pregnancy rate were also analyzed based on the time at which the embryo transfers were performed: cleavage stage (day 2/3) and/or blastocyst stage (day 5/6). There were no significant differences between single and sequential medium for clinical pregnancy (RR=1.09; 95%CI=0.83-1.44; *p*=0.53), ongoing pregnancy (RR=1.11; 95%CI=0.87-1.40; *p*=0.39), or miscarriage rates (RR=0.89; 95%CI=0.44-1.81; *p*=0.74). No significant difference was found for ongoing pregnancy rate (RR=1.29; 95%CI=0.93-1.78; *p*=0.12) between single and sequential medium when only trials in which embryos were transferred at the blastocyst stage were included. In conclusion, the choice of embryo culture approach - single or sequential medium - did not affect the ongoing pregnancy rates of patients undergoing ART cycles.

## INTRODUCTION

Several factors affect the success of *in vitro* fertilization (IVF) treatment. Proper embryo culture conditions and medium formulation in particular are two indispensable requirements (Mauri *et al.*, 2001; Summers & Biggers, 2003; Hentemann & Bertheussen, 2009; Paternot *et al.*, 2010). The fallopian tubes are the natural environment for human oocyte fertilization and the stage where early embryonic development unfolds; they provide the optimal environment for the developing preimplantation embryo before it reaches the endometrial cavity (Lyons *et al.*, 2006; Hambiliki *et al.*, 2011). Thus, the culture medium used in assisted reproductive technology (ART) procedures must be optimized to reproduce the environment in the fallopian tubes and the endometrium. Culture conditions play a determining role in embryo development and consequently in the achievement of ongoing pregnancy (Lonergan *et al.*, 2003; Hambiliki *et al.*, 2011). With that in mind, two possible formulations have been considered for culture media: one is the "back to nature" approach, based on sequential embryo culture medium (Sequential-ECM) designed to mimic *in vivo* conditions; and the other is the "let the embryo choose" approach, based on single culture medium (Single-ECM) in which the embryo is cultured in a constant medium containing all the ingredients needed for its development (Lane & Gardner, 2007; Biggers & Summers, 2008; Paternot *et al.*, 2010).

In view of the lack of data to define which approach provides for better embryo culture conditions, this meta-analysis aimed to find whether single medium was better than sequential medium in terms of clinical pregnancy, ongoing pregnancy, and miscarriage rates in patients undergoing ART procedures.

## MATERIAL AND METHODS

### Meta-analysis inclusion criteria

Inclusion criteria: published randomized controlled trials comparing the effectiveness of single and sequential medium at improving clinical outcomes among patients undergoing IVF/intracytoplasmic sperm injection (ICSI) cycles; trials providing data on IVF cycles.

### Endpoints

The primary endpoint measured in this meta-analysis was ongoing pregnancy rate (per randomized patient). The secondary endpoints included clinical pregnancy rate (per randomized patient) and miscarriage rate (from clinical pregnancy). Clinical pregnancy was defined as the presence of a gestational sac in the uterine cavity (with or without fetal heartbeat) at 6/7 weeks of gestation detected by ultrasound examination. Ongoing pregnancy was defined as the presence of fetal heartbeat at 6 to 12 weeks of gestation. Miscarriage was defined as any pregnancy, including clinical pregnancies, which failed to reach the status of ongoing pregnancy.

### Study selection

A search was carried out on electronic databases (PubMed, EMBASE, Web of Science, SCOPUS, and Cochrane Central Register of Controlled Trials; keywords: single culture medium, single step, sequential culture medium, continuous culture medium, pregnancy rates) to find randomized controlled trials published by December 2016 in which ART outcomes were compared vis-à-vis the use of single or sequential medium. The search included only papers published in English. The following headings and descriptors were used: IVF, ICSI, ART procedures, embryo culture media, sequential culture medium, single culture medium, clinical outcomes, and randomized study. Only randomized controlled trials were included.

### Search results

**Figure 1.**
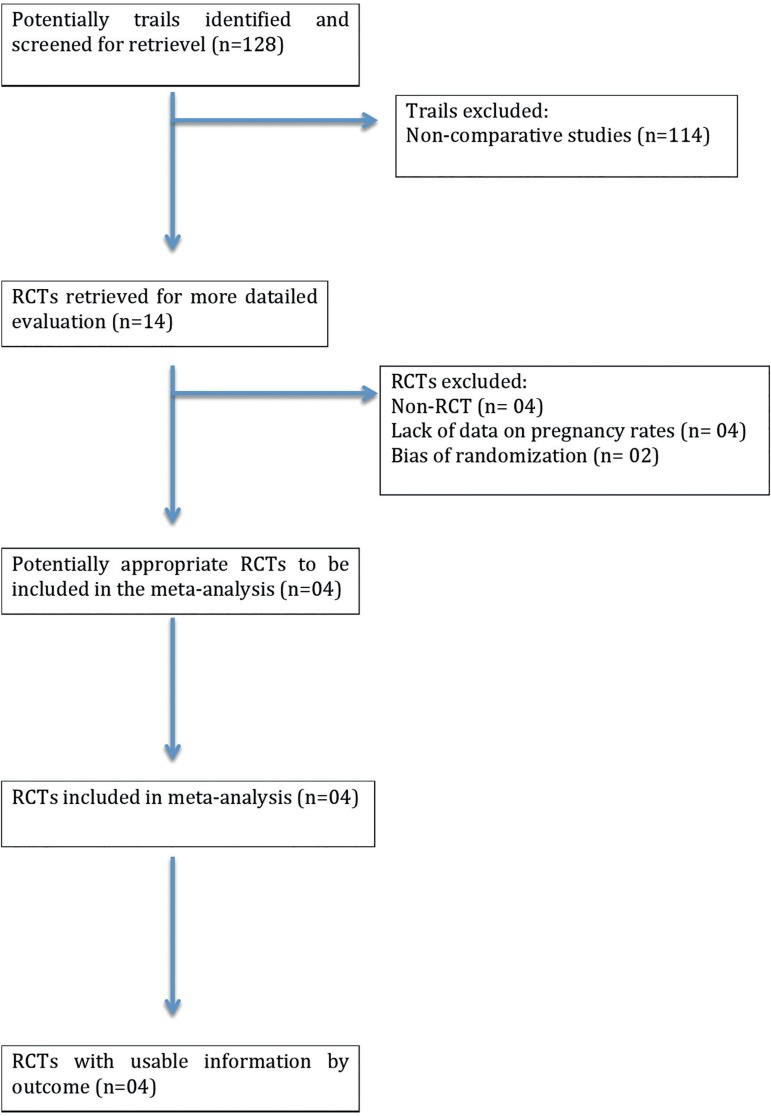
QUOROM statement flow diagram illustrating the selection of trials included in this meta-analysis.

Four of 128 trials met the inclusion criteria (Macklon *et al.*, 2002; Sepúlveda *et al.*, 2009; Campo *et al.*, 2010; Sfontouris *et al.*, 2015). Figure 1 shows a flow diagram depicting the selection process.

### Validity assessment and data extraction

Each trial was assessed independently by four reviewers (FCD, JBAO, RLRB, and JGFJr) and ranked based on methodological rigor and potential introduction of bias. They were analyzed for originally reported characteristics, randomization method, statistical power calculation, unit of analysis, and presence or absence of blinding. Missing data were obtained from the authors.

### A brief description of the included studies

Macklon *et al*., 2002: this prospective study compared blastulation, implantation, and viable pregnancy rates from embryos randomized to culture in sequential or single medium. Embryos obtained from patients submitted to IVF were randomized in sealed envelopes to culture in one of three systems: monoculture medium (Rotterdam culture media), the same culture medium with refresh on day three of embryo development, or sequential medium (Vitrolife G1 until day 3 and Vitrolife G2 until day 5). The blinding procedure was not described. The analysis included 146 treatment cycles (98 randomized to single medium and 48 to sequential medium). No significant differences were reported in ongoing pregnancy rates between the groups. Patient baseline characteristics were similar between the two groups (i.e. age). The authors indicated that human embryos do not necessarily have to be cultured in specifically designed sequential media to fully develop into viable blastocysts capable of resulting in ongoing pregnancy.

Sepúlveda *et al.*, 2009: this prospective randomized study used donor oocytes to compare single medium (Global medium) refreshed on day 3 and sequential medium (ECM/Multiblast) for the development of human embryos to the blastocyst stage and subsequent pregnancy outcomes. A total of 79 patients were randomly assigned to have their embryos cultured in single (40) or sequential medium (39). For clinical reasons, the pregnancy data of one of the patients was not included in statistical analysis. Patient baseline characteristics were similar between the two groups (i.e. age). Ongoing pregnancy rates tended to be higher after culture in single medium than in sequential medium, but the difference was not statically significant.

Campo *et al.*, 2010: this prospective randomized trial compared sequential (ISM1) and single medium (GM501) for pregnancy outcomes. All 172 patients seen in a four-month period in a human reproduction center for IVF/ICSI treatment were randomly allocated to either single (84) or sequential medium (83) cultures. Randomization was blinded by means of envelopes with an equal amount of stickers printed either single or sequential medium. Conventional embryo transfer was performed on day 2 or 3 (cleavage stage). Patient baseline characteristics were similar between the two groups (i.e. age). No differences between the two groups with regard to any of the outcomes analyzed (i.e., clinical pregnancy rate, pregnancy losses), including ongoing pregnancy rate, were reported. In conclusion, there was no significant difference between sequential and single medium cultures.

Sfontouris *et al.*, 2015: This randomized controlled trial (RCT) included 100 patients randomly allocated to embryo culture in either single [Global medium (50 patients)] or sequential medium [Origio-ISM1/BlastAssist (50 patients)] within a six-month period. Physicians and patients were blinded to the type of medium used. Embryo transfers were performed on day 2, 3 or 5. Patient baseline characteristics were similar in the two groups (i.e. age). No statistically significant differences, including, were found between single and sequential medium - ongoing pregnancy rates of [50% (22/44) versus 50% (24/48)] respectively. Both culture approaches (single and sequential medium) appear to adequately support *in vitro* pre-implantation embryo development and produce similar reproductive outcomes.

### Statistical analysis

Four RCTs were included as targets for data extraction and meta-analysis. Data were combined for meta-analysis using statistical package Stats-Direct. Dichotomous variables were expressed in the form of Relative Risk (RR) with a 95% confidence interval (CI). Heterogeneity was assessed using Cochran's Q and I ^2^. Study data were combined using a Random-effects model. *p*-values <0.05 were considered statistically significant.

## RESULTS

This meta-analysis looked into clinical pregnancy (per randomized patient), ongoing pregnancy (per randomized patient), and miscarriage (from clinical pregnancy) rates. Ongoing pregnancy rates were shown based on the time of embryo transfer: cleavage stage (day 2/3) and/or blastocyst stage (day 5/6) of embryo development.

**Figure 2.**
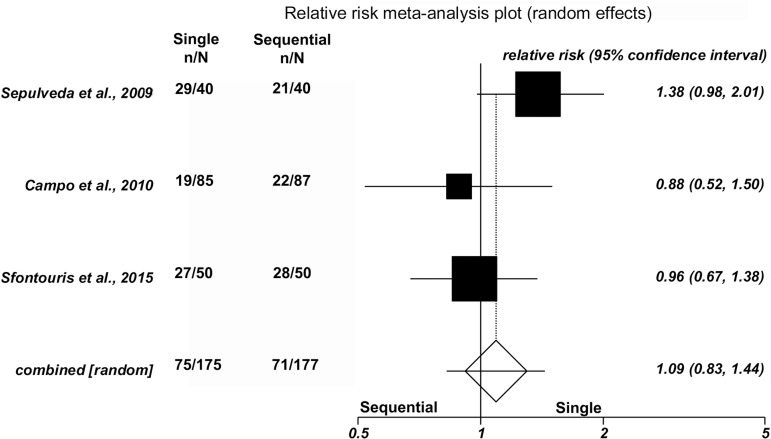
Forest plot - clinical pregnancy rate per randomized patient.

**Figure 3.**
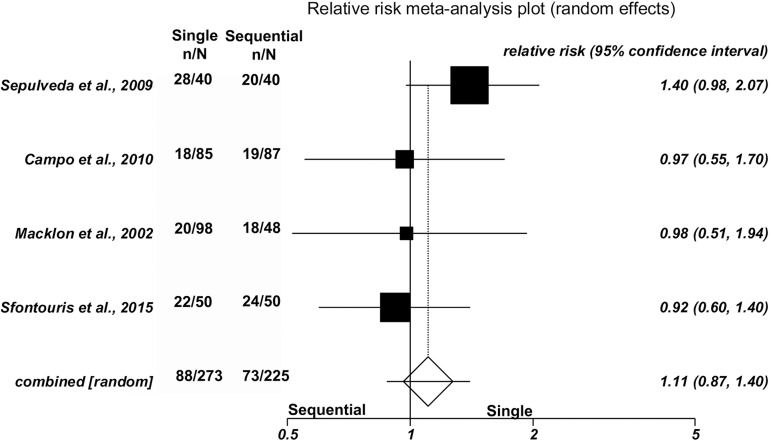
Forest plot - ongoing pregnancy rate per randomized patient.

**Figure 4.**
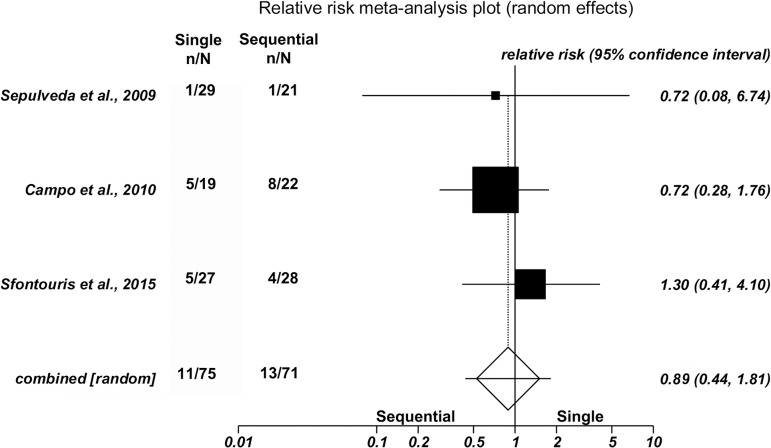
Forest plot - miscarriage rate from clinical pregnancy.

No significant differences were found between single and sequential medium for clinical pregnancy in three studies [42.9% (75/175) versus 40.1% (71/177), (RR=1.09; 95%CI=0.83-1.44; *p*=0.53/Heterogeneity: Cochran Q=2.88, *p*=0.23, I^2^=30.5%)]; ongoing pregnancy in four studies [32.2% (88/273) versus 32.4%(73/225), (RR=1.11; 95%CI=0.87-1.40; *p*=0.39/Heterogeneity: Cochran Q=2.72, *p*=0.44, I^2^=0%)]; or miscarriage rates in three studies [14.7% (11/75) versus 18.3% (13/71), (RR=0.89; 95%CI=0.44-1.81; *p*=0.74/Heterogeneity: Cochran Q=0.59, *p*=0.75, I^2^=0%)]. Forest plots depict the results of the individual studies included in this meta-analysis (Figures 2, 3 and 4).

No significant differences were found between single and sequential medium in the RCTs in which embryo transfer was carried out at the blastocyst stage [Single-ECM: 34.8% (48/138) *versus* Sequential-ECM: 34.1% (30/88), (RR=1.29; 95%CI=0.93-1.78; *p*=0.12/Heterogeneity: Cochran Q=0.90)]. Table 1 describes the ongoing pregnancy rate results according to the stage of embryonic development at the time of embryo transfer.

**Table 1:** Single medium versus sequential medium. Ongoing pregnancy rate outcomes. Random effects.

Trial	Single-ECM n/N	Sequential-ECM n/N	RR	95%CI
**Cleavage stage**				
Campo *et al*., 2010	18/85	19/87	0.97	0.55-1.70
**Blastocyst stage**				
Sepulveda *et al*., 2009	28/40	20/40	1.40	0.98-2.07
Macklon *et al*., 2002	20/98	10/48	0.98	0.51-1.94
**Cleavage and Blastocyst stages**				
Sfontouris *et al*., 2015	22/50	24/50	0.92	0.60-1.40
**Total (Random effects) combined**	88/273	73/225	1.11	0.87-1.40
**Blastocyst stage only (Random effects) combined**	48/138	30/88	1.29	0.93-1.78
**Total** Chi2=0.71 *p*=0.39 Cochran Q=2.72 *p*=0.44 I^2^=0% (95% CI=0% to 67.9%)		**Blastocyst stage only** Chi2=2.35 *p*=0.13 Cochran Q=0.90 *p*=0.34		

## DISCUSSION

Meta-analyses often shed light on medical questions surrounded by uncertainty. This method of analysis integrates and combines the findings of various independent studies into a single common result. Compared to narrative reviews, meta-analyses are less biased by a reviewer's personal opinions, and are thus less likely to provide partial conclusions. Additionally, the results may be easily recalculated and compared to the conclusions stated by the authors. Several human embryo culture procedures have been proposed to improve the clinical outcomes of patients submitted to ART, and two approaches stand out from the crowd: single and sequential medium (Reed *et al.*, 2009; Paternot *et al.*, 2010). This meta-analysis compared single and sequential medium for how each performed in terms of the clinical outcomes of patients submitted to ART, and found that the two methods did not produce statistically different outcomes.

Embryo culture is an important step in ART, and culture medium plays a key role in the success of IVF cycles (Yoon *et al.*, 2011; Khoury *et al.*, 2012). An ideal embryo culture medium should contain components similar to the ones present in the natural environment, to thus mimic an environment most conducive to pre-implantation embryonic development and enable improved clinical outcomes (Campo *et al.*, 2010). With this idea in mind, sequential medium with the "back to nature" approach was developed to mimic the changing embryo development environment, first in the Fallopian tubes and then in the uterus, in which different formulations are required (Gardner, 1998; Gardner & Lane, 2014; Hardarson *et al.*, 2015). On the other hand, single medium with the "let the embryo choose" approach was designed to meet the metabolic needs of both early and late-stage embryo development during *in vitro* culture; therefore, this medium must contain all ingredients needed for the embryo to develop to the blastocyst stage (Campo *et al.*, 2010; Hambilik *et al.*, 2011; Machtinger & Racowsky, 2012; Quinn, 2012). Several authors have compared the clinical outcomes resulting from different types of embryo culture medium, single and sequential medium (Sepúlveda *et al.*, 2009; Campo *et al.*, 2010; Macklon *et al.*, 2002; Alteri *et al.*, 2015; Hardarson *et al.*, 2015; Scarica *et al.*, 2015; Sfontouris *et al.*, 2015; Sfontouris *et al.*, 2016), but despite the numerous publications on the matter, some studies had methodological flaws such as randomization bias, unclearly measured outcomes, inadequate sample sizes, and absence of statistical power calculation.

The single medium approach may, in many ways, be advantageous in laboratory practice. This method requires less embryo handling and thus minimizes the chances of unintentionally damaging the embryo, considering that regardless of the chosen culture system, embryos have to be frequently removed from the incubator to have their development assessed, thus exposing them to stress and reactive oxygen species. Single medium also provides adequate stabilization of the embryo culture environment and reduces global costs (Gardner & Lane, 1996; Selvakumar *et al.*, 2006; Xie *et al.*, 2007; Machtinger & Racowsky, 2012; Hardarson *et al.*, 2015). In addition, single medium is also a friendlier method when one uses time-lapse systems. This explains why single medium is growing in popularity, since time-lapse systems may prevent repeated disturbances to the embryonic culture environment. However, it should be noted that this method of embryo selection is not yet established in most human reproduction centers (Ciray *et al.*, 2012; Hardarson *et al.*, 2015).

In conclusion, single and sequential medium did not produce significantly different outcomes in terms of clinical pregnancy, ongoing pregnancy, or miscarriage rates in patients undergoing ART cycles. Although no differences were found between the two embryo culture methods, this meta-analysis included only a few randomized clinical trials and a small number of patients. Therefore, inferences from the findings described herein must be made with caution.
